# Influence of Culture Conditions on In Vitro Asymbiotic Germination of *Anacamptis longicornu* and *Ophrys panormitana* (Orchidaceae)

**DOI:** 10.3390/plants10112543

**Published:** 2021-11-22

**Authors:** Myriam Arcidiacono, Caterina Catalano, Antonio Motisi, Maurizio Sajeva, Francesco Carimi, Angela Carra

**Affiliations:** 1 Institute of Life Sciences, Scuola Superiore Sant’Anna, Piazza Martiri della Libertà 33, 56127 Pisa, Italy; Myriam.Arcidiacono@santannapisa.it; 2 Institute of Biosciences and BioResources, National Research Council, Corso Calatafimi 414, 90129 Palermo, Italy; antonio.motisi@ibbr.cnr.it (A.M.); francesco.carimi@ibbr.cnr.it (F.C.); angela.carra@ibbr.cnr.it (A.C.); 3 Department of Biological, Chemical and Pharmaceutical Sciences and Technologies, University of Palermo, Viale delle Scienze, 90128 Palermo, Italy; maurizio.sajeva@unipa.it

**Keywords:** acclimatization, embryonic developmental stages, endangered species, Mediterranean terrestrial orchids, photoperiod

## Abstract

This study is the first approach to in vitro asymbiotic germination of two species of Sicilian threatened terrestrial orchids, *Anacamptis longicornu* and *Ophrys panormitana.* Seeds were collected in the wild and cultured in two different media—Orchimax medium (OM) and Murashige and Skoog (MS)—and exposed to different photoperiods and temperatures to evaluate the best conditions for the specific stages of development. The germination of *A. longicornu* was very high on OM (95.5%) and lower on MS medium (21.4%), whereas *O. panormitana* germinated only on OM medium, with significantly lower percentages (12.0%), compared with *A. longicornu*. This difference is caused by variation in quality and quantity of nutrients used, primarily by nitrogen source. The results show that temperature and photoperiod widely affect seed germination and development. Although further investigations on asymbiotic and symbiotic germination are needed for the improvement of conservation of Mediterranean terrestrial orchids, our results contribute to the conservation of this group of plants.

## 1. Introduction

Family Orchidaceae includes about 25,000 species distributed in more than 800 genera and, along with Asteraceae, is one of the two largest families of higher plants [1].

The broad geographical distribution, the wide richness of species, and the genetic diversity distinctive of this family are matched with its rarity; most orchid species are threatened or endangered in the wild mainly due to habitat alteration or destruction, over-harvesting, and illegal trade [2,3]. Orchids are used for multiple purposes such as medicines, food [4,5], and cosmetic products [6,7]. Usually, the supply is from wild-collected plants [6]. All orchid species are listed in the Convention on International Trade in Endangered Species (CITES) of Wild Fauna and Flora Appendices, and the European Union has issued more restrictive regulations for trade (https://www.cites.org/eng/app/appendices.php, accessed date 1 September 2021). In situ protection and management of isolated and poor populations is not enough for species survival [8] and, for this reason, ex situ and in situ conservation programs are essential to safeguard orchid germplasm [9,10]. This is particularly true for European terrestrial orchids; they have a specialized niche of collectors and are used in the cosmetic and food industry [11]. In these cases, the origin of plants is always wild [12].

Endangered plants produced in vitro improve the conservation efforts, providing commercial material and lowering the pressure on the habitat. Plant biotechnologies allow rapid multiplication of genetically homogeneous plants in reduced spaces; they are very useful for species that could not be propagated otherwise or for which the propagation is problematic, enabling the production of virus-free plants; these techniques are useful for the production of plants from seeds with low chances of successful germination and growth in the field. This is the case with terrestrial orchids. The absence of enzymes to metabolize polysaccharides and lipid forces, cause orchids to have a mycorrhizal relationship with compatible fungi during germination and early development [13]. Mycobionts penetrate into the base of the embryo, at the earliest stages of germination, and supply water, vitamins, minerals, and carbohydrates. Mycobionts are therefore necessary for the germination of these orchids. This aspect makes it difficult to obtain seedlings, as researchers must find the specific mycobiont to allow germination.

Some techniques of symbiotic micropropagation have been developed to isolate and cultivate mycobionts harvested from wild orchids and consequently to inoculate in vitro orchid seeds with these fungi [14]. However, successful germination can involve pairing the orchid species with the correct and appropriate strain or species of fungus [15]. In vitro fungal specificity seems to be very high for some species, and terrestrial orchids appear to have a higher degree of mycobiont specificity at the generic and species levels [16]. Consequently, mass propagation of terrestrial species from seeds can be a very difficult proposition [2]. For this reason, in vitro asymbiotic germination of orchid seeds on agar culture media with different combinations of nutrients is sometimes preferred. Moreover, using seeds it is possible to obtain new individuals that are different from the mother plant and, in terms of biodiversity conservation, this feature is desirable.

Sicily is one of the regions of Italy with the highest incidence of orchids, with 90 taxa, including species and subspecies [17]. The aim of this work was to attempt, for the first time, in vitro asymbiotic germination of *Anacamptis longicornu* and *Ophrys panormitana*, two species of wild terrestrial Sicilian orchids. The possibility to obtain artificially propagated specimens may take the pressure off wild populations and, at the same time, may supply valued material both for collectors and food and cosmetic industries.

**Figure 1:**
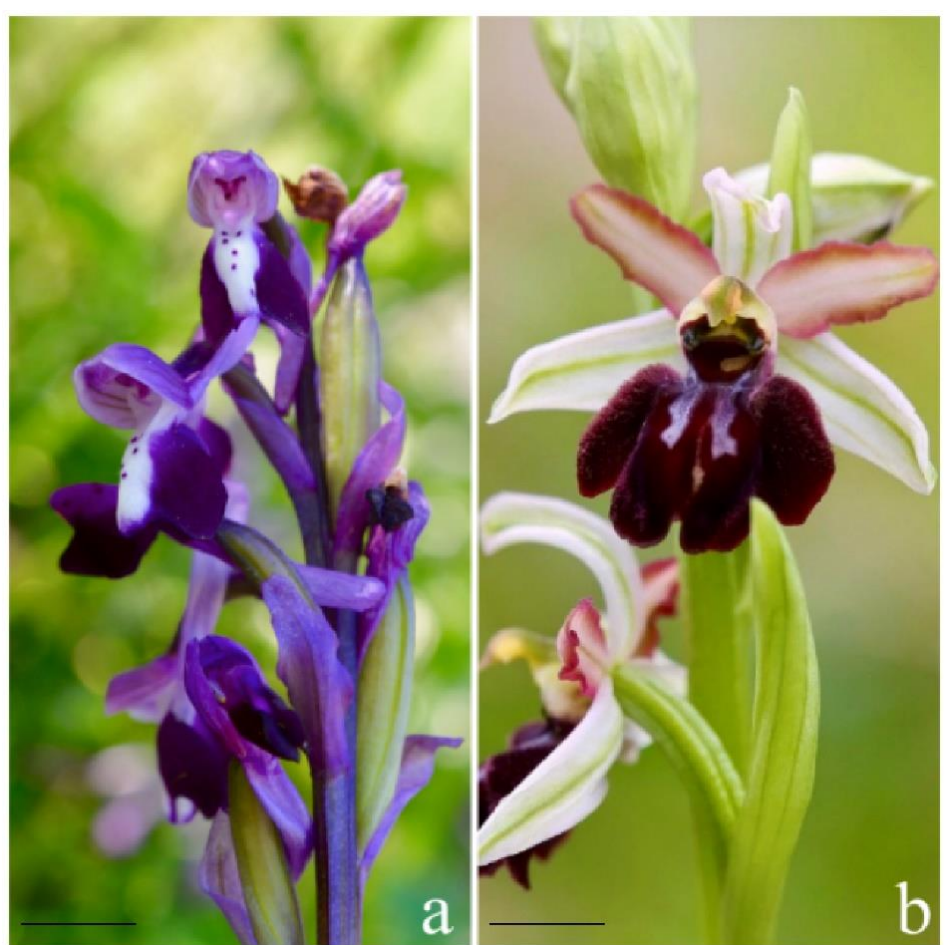
(**a**) Specimen of *Anacamptis longicornu* growing on Pizzo Neviera (PA); (**b**) *Ophrys panormitana*. (Photo: Amata Ciro). Bars: 8 mm (**a**); 5 mm (**b**).

The genus *Anacamptis* is spread in central Europe and in Mediterranean countries, expanding to Caucasus and Iran. About 30 entities, including 8–10 taxa in Italy, belong to this genus. *A. longicornu* R.M. Bateman, Pridgeon & M.W is generally an orchid of small size, with a height of 10–35 cm; the root system has two almost rounded tubers. It blooms between the end of March and May (Figure 1a). With regard to the habitat, it is not a demanding species concerning the composition of the substrate, and it is possible to find it at altitudes ranging from 0 to 1200 m and in various habitats, including scrublands, garrigues, oligotrophic grasslands, glades of woodlands, and abandoned croplands, in bright sunshine or half-shade *A. longicornu* displays a western steno-Mediterranean distribution type; it is present and widespread in Sardinia and Sicily. Its presence in continental Italy has not been confirmed yet. It has also been recorded in Corsica, in the Balearic Islands, and in North African countries [18].

The genus *Ophrys* is spread in Mediterranean environments, mainly European. The number of species decreases significantly at increasing altitudes and latitudes. *O. panormitana* (Tod.) Soó is a robust plant, with a height of 15–40 cm and flowers of medium size. It blooms between December and March (Figure 1b). It grows on oligotrophic grasslands, garrigues, glades of woodlands, even in the shade, and it thrives up to 1200 m of altitude. It is an endemic species of Sicily, especially on the NW and SE of the island [19].

## 2. Materials and Methods

### 2.1. Plant Material, Seed Collection, and Axenic Culture Establishment

Seeds of *A. longicornu* were collected from “Pizzo Neviera” (38°02′29″ N, 13°23′22″ E) from near mature capsules (slightly yellow) on different plants. Two mature capsules (with fissures on the surface) of *O. panormitana* were collected from a site near Palermo (Barcarello: 38°12′50.09″ N 13°17′36.04″ E). Seeds were stored at 4 ± 1 °C before sowing.

Intact capsules, with no holes or fissures over it, were disinfected under laminar flow with ethanol 70% for 1 min and commercial bleaching solution (with 2.2% active chlorine) 20% for 5 min, followed by three rinses with sterile distilled water for 5 min each.

**Figure 2:**
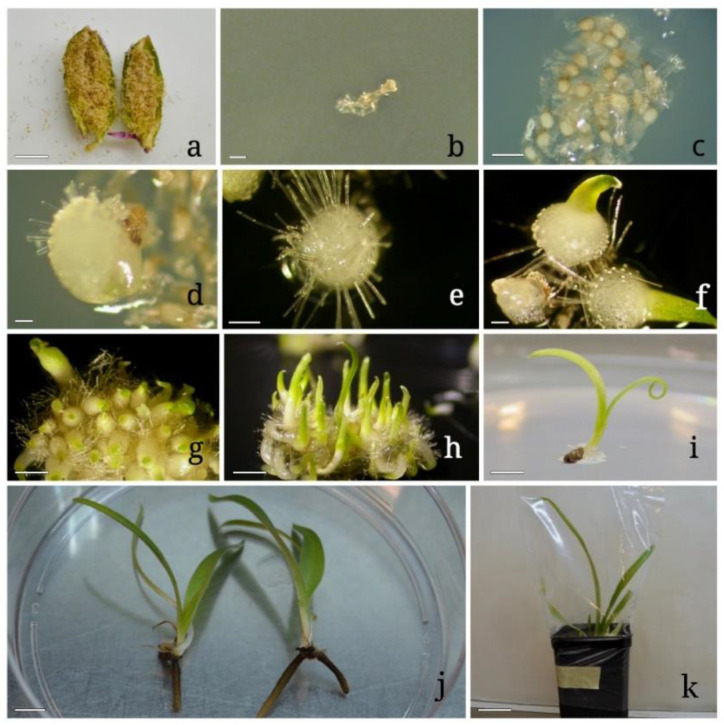
In vitro asymbiotic germination of *Anacamptis longicornu*: (**a**) opened capsule of *Anacamptis longicornu*; (**b**) stage 0 ‘’no germination’’; (**c**) stage 1 ‘’pre-germination’’; (**d**) stage 2 ‘’germination’’; (**e**) stage 3 ‘’protocorms’’; (**f**) stage 4 ‘’appearance of protomeristem’’; (**g**) stage 5 ‘’emergence of first leaf’’; (**h**) stage 6 ‘’elongation of first leaf’’; (**i**) stage 7 ‘’emergence of second leaf’’; (**j**) emergence of first true root; (**k**) acclimatization. Bars: 10 mm (**a**,**h**–**k**); 2 mm (**b**–**d**); 3 mm (**e**,**f**); 0.5 mm (**g**).

**Figure 3:**
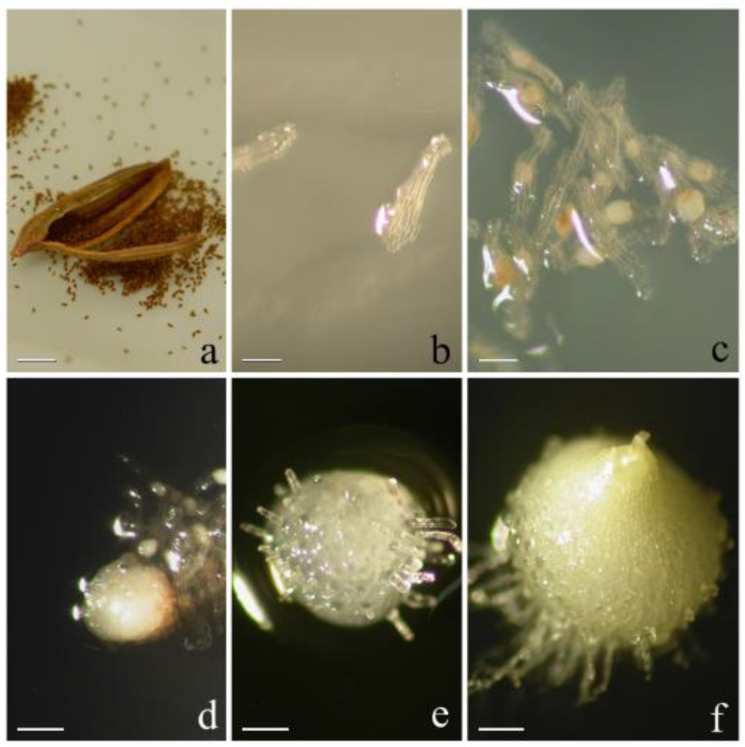
In vitro asymbiotic germination of *Ophrys panormitana*: (**a**) opened capsule of *Ophrys panormitana*; (**b**) stage 0 ‘’no germination’’; (**c**) stage 1 ‘’pre-germination’’; (**d**) stage 2 ‘’germination’’; (**e**) stage 3 ‘’protocorms’’; (**f**) stage 4 ‘’appearance of protomeristem’’. Bars: 5 mm (**a**); 1 mm (**b**,**c**); 4 mm (**d**–**f**).

The sterilization of seeds from mature open capsules (Figure 2a and Figure 3a) was slightly reduced: ethanol 70% for 30 s and commercial bleach 20% for 2 min.

### 2.2. Culture Conditions

For axenic culture establishment, we used Petri dishes 60 mm × 15 mm, sealed with Parafilm M^TM^. Seeds were incubated on two different basal media, Orchimax Medium (Duchefa Biochemie, Haarlem, The Netherlands) (OM), with activated charcoal and 30 g/L of sucrose, and Murashige and Skoog [20] medium (Duchefa Biochemie, Haarlem, The Netherlands) (MS), with 20 g/L of sucrose, differing mainly in the total concentration of micro and macro elements and in the nitrogen source (organic and inorganic in the OM, only inorganic in the MS medium). Both media were solidified with 7 g/L of Plantagar (S1000, B&V srl Borgo Regale, Parma, Italy) and the pH was adjusted to 5.6 ± 0.1 with 0.5 M KOH before autoclaving at 121 °C and 1 atm for 20 min.

Seeds were exposed to different photoperiods and temperatures, evaluated for the different specific developmental stages. Seeds, when incubated in the light, were exposed to a cool white fluorescent lamp, with a photosynthetic photon flux density of 50 µmol m^−1^ s^−1^. In the beginning, plates were incubated in a growth chamber under a long-day (LD) photoperiod (16/8 h at 25 ± 1 °C. Then, 45 days after sowing, seeds were exposed to neutral-day (ND) photoperiod (12/12 h) at 18 ± 1 °C to simulate the typical autumn conditions, and after 120 days from sowing, the seeds were transferred back to the initial conditions. After about 6 months from sowing in MS medium and about 10 months from sowing in OM, plantlets were transferred in sterile magenta boxes containing 50 mL MS medium. After about 18 months from sowing, all the plants obtained were transferred at 18 ± 1 °C under ND photoperiod.

The scheme of the procedure is reported in Table 1.

### 2.3. Germination and Protocorm Formation

The process of germination and development of orchid seeds was evaluated according to the embryonic developmental stage (Table 2), modified from Stewart and Zettler [21].

**Figure 4:**
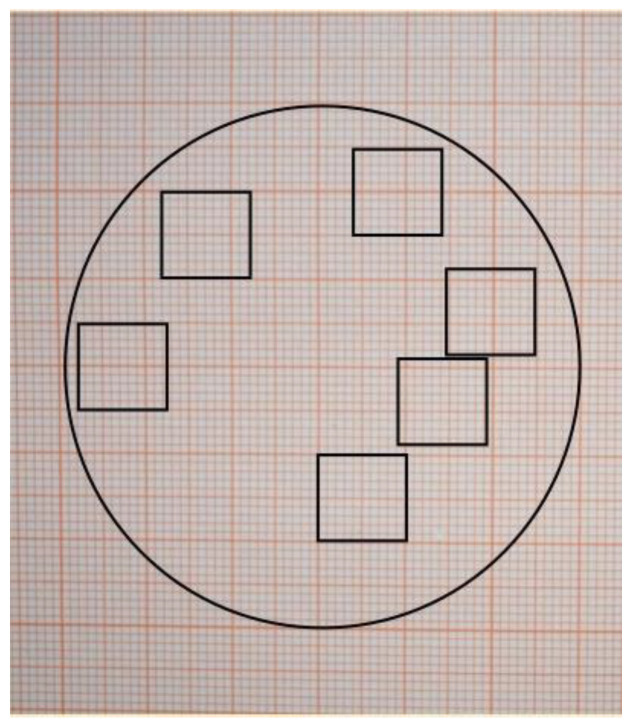
Grids used to count seeds and seedlings.

The germination percentages were calculated using a procedure similar to the evaluation of red blood cell count with a Burker hemocytometer counting grid. A grid with squares of 1 cm^2^ was drawn on transparent paper, which was placed on the Petri dishes culture, and 6 squares were randomly chosen (Figure 4). The number of germinated and not-germinated seeds was counted for each square using a stereomicroscope.

The estimate of germinated seeds was obtained by multiplication of the total number counted on all the squares by culture plate area and dividing the result by 6 cm^2^. The percentages were calculated using the proportion with the total number of seeds for each plate.

### 2.4. Plant Acclimatization

Plantlets, 10–15 cm high and with well-developed roots, were transferred from MS medium to acclimatization. Once roots were washed with distilled water to remove agar residues, plantlets were transferred into 10 cm diameter pots containing a sterilized mixture of orchids loam mixed with a pumice stone. The pots were covered with a transparent polyethylene bag and placed in a climate chamber at 18 ± 1 °C under ND photoperiod, and a photosynthetic photon flux of 50 μmol m^−2^ s^−1^ was provided with Osram cool-white 18 W fluorescent lamps. After 8 weeks under these conditions, the transparent polyethylene bag was gradually perforated. The acclimation bags were definitively removed after 4 weeks, and the plants were transferred outdoor under natural daylight conditions. The survival rate was recorded after 2 months.

## 3. Results

Germination occurred for both species after exposure to ND photoperiod. Results will be discussed in detail analyzing the two species separately.

### 3.1. Anacamptis Longicornu

When exposed to LD photoperiod and 25 ± 1 °C the totality of seeds remained in stage 0 (Figure 2b), with no changes in the aspect. After transfer to ND photoperiod and 18 ± 1 °C, all the seeds reached stage 1 of “pre-germination”, in which embryos are swollen, in a few days (Figure 2c).

**Figure 5:**
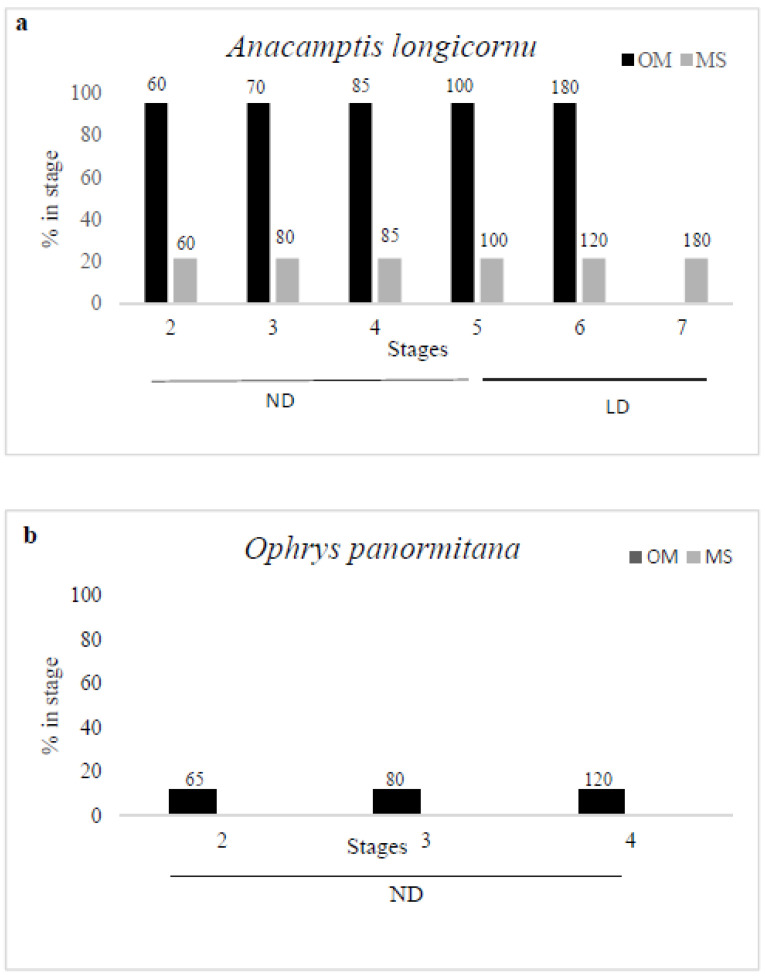
Effect of culture medium and photoperiod on in vitro seedling development stage of *Ancamptis longicornu* (**a**) and *Ophrys panormitana* (**b**). Numbers on columns indicate days after sowing. ND—neutral day; LD—long day.

About 60 days after sowing, seeds germinated reaching stage 2 (Figure 2d), in which embryo emerges from the seed coat, with percentages ranging from 95.5% on OM to 21.4% on MS medium. Germinated seeds reached stages 3 (Figure 2e), 4 (Figure 2f), and 5 (Figure 2g) of development, respectively, 70, 85, and 100 days from sowing on OM and 80, 85, and 100 days from sowing on MS medium, maintaining the same percentages (Figure 5a). Seedlings increased steadily in size, reaching stage 6 (Figure 2h) (elongation of the first leaf) 120 and 150 days after sowing on MS medium and on OM, respectively. Seedlings on MS Medium reached stage 7 (emergence of the second leaf) about 180 days after sowing. When plants on MS medium reached stage 7 (Figure 2i), they were transferred in sterile magenta boxes on the same medium. Under this culture condition, plants increased steadily in size, reaching the height of about 5 cm in 2 months. One year after sowing, the presence of 73 plants with functional roots was recorded (Figure 2j). After 18 months from sowing, all plants were moved to a growth chamber under ND photoperiod and 18 ± 1 °C to enhance their development.

### 3.2. Ophrys Panormitana

No significant changes were recorded on both culture media when seeds were incubated under LD photoperiod and 25 ± 1 °C (Figure 3b).

About 60 days from sowing, the totality of seeds reached stage 1 on both media (Figure 3c). The phase of germination (stage 2, Figure 3d) occurred 65 days after sowing on OM, with percentages of 11.8%; protocorms (stage 3, Figure 3e) were formed 80 days after sowing, while seeds reached stage 4 (Figure 3f) with the appearance of protomeristems, 120 days after sowing. After the appearance of protomeristems, development ceased on OM.

On MS medium, only some seeds reached stage 2, breaking the testa of the embryo. Thereafter, the process ceased on this medium (Figure 5b).

### 3.3. Plant Acclimatization

During the first period of acclimatization, about 90% of plantlets transferred on pots grew vigorously. After the transparent polyethylene bag was perforated, the percentage of plantlet survival decreased to 60%. Two months after the acclimation bags were definitively removed, a survival rate of 30% was recorded (Figure 2k).

## 4. Discussion

Terrestrial orchids for germination are dependent on symbiosis with fungi in nature. When asymbiotic germination is attempted, several factors contribute to the final result. Timing of seed collection [22,23], composition of culture medium, [16,24,25], light intensity, temperature ranges [26] and seed pretreatments [27] contribute to successful germination. In this study, we analyzed the effect of temperature, photoperiod, and culture media composition on asymbiotic orchids germination [28].

Our results demonstrate that temperature and photoperiod widely affect seeds germination and seedling development. For terrestrial orchids, germination rate is dependent on temperature [28], but its role during orchid seed germination and seedling development has been largely ignored by the researchers, especially because photoperiod is often considered more important than temperature [29]. However, for many plant species, the temperature is a major factor responsible for the onset and breaking of physiological seed dormancy [30]. Since germination responses to photoperiods are often species specific, the growing conditions in situ should be considered when determining an appropriate photoperiod [29,31]. Baskin et al. [32] recommended alternating temperature regimes for studying the germination ecology of all seeds, as constant temperatures are not common in nature. For this reason, throughout the study, temperature and photoperiod were chosen to try to mimic the natural conditions in which Sicilian orchids grow. As no changes in seeds development were observed under the LD photoperiod both for *A. longicornu* and *O. panormitana*, plates were moved under ND photoperiod conditions. A few days after the transfer, seeds of both species reached stage 1 and, subsequently, the following stages of germination until the emergence of the first leaf. These data are in contrast with those reported by Kauth et al. [29], who achieved the highest percentage of germination under short-day conditions for seeds coming from Florida. In our experimental system, ND photoperiod was chosen to stimulate germination because Sicilian orchids germinate in nature in autumn when the temperature is around 18–20 °C, and water is more available compared with the typical dryness of the Mediterranean climate in the summer months.

**Figure 6:**
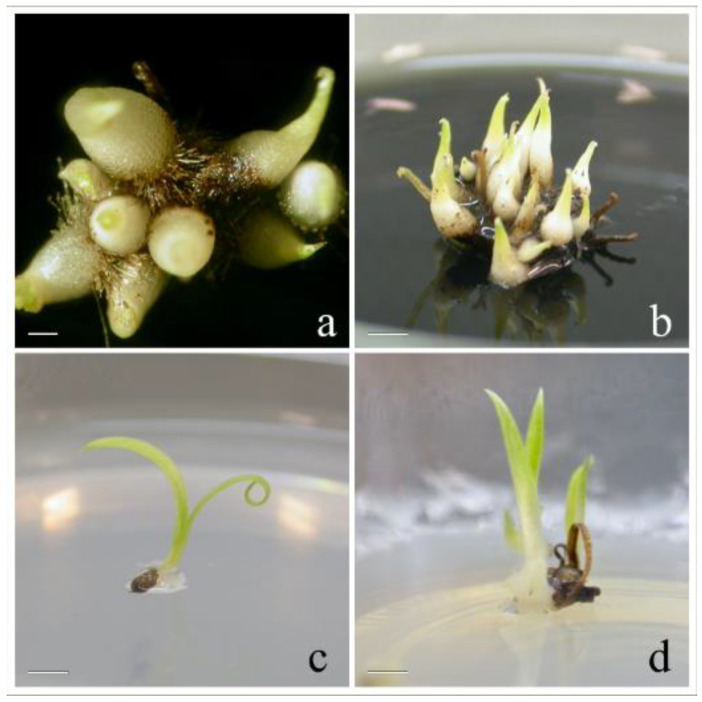
Different growth patterns of *Anacamptis longicornu* seedlings in the two culture media: (**a**,**b**) seedlings on OM medium; (**c**,**d**) seedlings on MS medium.

The choice of culture medium strongly influences seed germination [33,34]. Differences in the composition of organic and inorganic nutrients significantly affect the final result [35,36,37,38]. With regard to *A. longicornu*, germination occurred on both culture media with significant differences in terms of percentage of germination (Figure 5). However, once seeds reached stage 5, with the emergence of the first leaf, development slowed down significantly. The elongation of the first leaf on MS occurred earlier and the seedlings, 190 days after sowing, reached stage 7 with the emergence of the second leaf. In addition, a different pattern was observed for *A. longicornu* seedlings in the two culture media (Figure 6). The OM induced protocorms broader and larger, while the first leaf grew barely and slowly. In MS medium, instead, the bottom of protocorms did not grow very much, but a rapid elongation of the first leaf occurred, followed by the emergence of the second leaf [39].

Some authors reported that, while low salt culture media may support initial germination, a different medium may better support subsequent development [29,39,40]. Having observed the different growth patterns between the two culture media, and since seedlings on OM took longer to reach stage 7, all plants were transferred to MS medium, with the hypothesis that this medium could positively influence the development of plantlets due to its high nutrient content, as reported also for other species [39]. After this transfer, plantlets reached quickly the next stage with the emergence of the first true root. These data supported the hypothesis by [24] that MS medium was more suitable than OM for plants development after germination. The same trend was observed for *O. panormitana* (Figure 5). Seeds in MS medium reached the stage of pre-germination (stage 1) and then stopped to develop, while seeds in OM developed up to the appearance of protomeristem (stage 4). The difference in germination and seedling development is caused by variation in quality and quantity of nutrients [41]. The total concentration of micro and macro elements of MS medium is almost double, compared with those of OM medium, which is enriched in organic additives (tryptone, a mixture of amino acids) to provide an additional source of reduced organic nitrogen. Nitrogen source and availability influence the germination of different orchid species [38,42,43], and it is essential for plant growth and development [16]. To test asymbiotic seed germination of four *Anacamptis* species, Magrini et al. [44] compared several culture media differing mainly in the nitrogen source, organic or inorganic: the medium BM-1 with only amino acids as nitrogen source promotes germination and supports seedlings growth. Similar results have been obtained by Dulić et al. [45] on *Ophrys sphegodes*, confirming that asymbiotic germination and protocorm formation is promoted by organic nitrogen. Moreover, our results confirmed the negative effects of inorganic sources of nitrogen (nitrate and ammonium salts), in accordance with previous studies on other species of temperate terrestrial orchids [46,47,48]. The low germination percentages of *A. longicornu* and the absence of germination of *O. panormitana* on MS medium could be attributed to high ammonium content and to the inability of the protocorms to use nitrates during the initial stages of development [49].

The different response in seed germination is influenced by media composition with nitrogen, among others, playing a central role in regulating the process. The low germination percentage in MS medium indicates an inhibitory effect due to the high salt concentration of macroelements [50]. Since terrestrial orchids usually require a medium with lower salt concentrations for seed germination [51], the OM medium fits this characteristic, as the total amount of macroelements is significantly lower than that present in the MS medium.

Seed germination of both *A. longicornu* and *O. panormitana* is faster, compared with that of other species reported in the literature [52,53]. Seeds of *A. longicornu* reached stage 2 of germination 60 days after sowing on both culture media, and seeds of *O. panormitana* reached this stage 65 days after sowing on OM medium. *O. palustris* [32] reached the same stage of germination 6 months after sowing on MS medium. Although seeds of *O. patens* [52] reached stage 2 of germination 45 days after sowing, the subsequent development of seeds was very slow. Seeds reached stage 3 just 120 days after sowing; the same stage of development was achieved earlier both in the case of *A. longicornu*, on OM 70 days after sowing and on MS medium 80 days after sowing, and in the case of *O. panormitana*, on OM 80 days after sowing. The homogeneity is also evident among germination percentages of the various stages. All the seeds that reached stage 2 subsequently achieved the following stages of development with the same percentages, in contrast with the results obtained by Magrini et al. [53], who reported a high level of heterogeneity between germination percentages.

To evaluate the influence of culture media on seed germination and seedling development, further studies are necessary. Moreover, a new investigation is needed to understand the mechanisms regulating in vitro orchid seed germination, especially because parameters for orchid seed germination seem to be species specific. Further studies on asymbiotic and symbiotic germination may be useful to improve the conservation strategy of Mediterranean terrestrial orchids.

## 5. Conclusions

In this manuscript, a procedure for in vitro asymbiotic germination of two species of Sicilian threatened terrestrial orchids, *Anacamptis longicornu* and *Ophrys panormitana*, was described. Several parameters influence seed germination. Seeds were cultured in two different media, OM and MS, and exposed to different photoperiods and different temperatures. The best performance was achieved with seeds cultured on OM medium, exposed mostly under LD photoperiod. Both genotypes respond positively producing new acclimatized plants. The establishment of a reproducible protocol for the germination of seeds of terrestrial orchid species is considered a prerequisite for their conservation and may be useful for safeguard programs.

## Figures and Tables

**Table 1:** Timing and culture conditions adopted for seed germination of *Anacamptis longicornu* and *Ophrys panormitana*.

Culture Conditions	Time from Sowing				
	0 Days	45 Days	120 Days	6 Months	10 Months
Medium	MS	MS	MS	MS	MS
	OM	OM	OM	OM	MS
Photoperiod	LD	ND	LD	LD	LD
Temperature	25 ± 1 °C	18 ± 1 °C	25 ± 1 °C	25 ± 1 °C	25 ± 1 °C

**Table 2:** Seedling developmental stages of *Anacamptis longicornu* and *Ophrys panormitana* [21].

Stage	Description
0	No germination
1	Pre-germination
2	Germination
3	Protocorms
4	Appearance of protomeristem
5	Emergence of first leaf
6	Elongation of first leaf
7	Emergence of second leaf

## Data Availability

The data presented in this study are available in the article.
